# SGA-WZ: A New Strapdown Airborne Gravimeter

**DOI:** 10.3390/s120709336

**Published:** 2012-07-06

**Authors:** Yangming Huang, Arne Vestergaard Olesen, Meiping Wu, Kaidong Zhang

**Affiliations:** 1 College of Mechatronics Engineering and Automation, National University of Defense Technology, Changsha 410073, Hunan, China; E-Mails: meipingwu@263.net (M.W.); kdzhang@263.net (K.Z.); 2 National Space Institute, Technical University of Denmark, Juliane Maries Vej 30, DK-2100, Copenhagen, Denmark; E-Mail: avo@space.dtu.dk

**Keywords:** airborne gravimeter, strapdown inertial navigation system, differential GPS

## Abstract

Inertial navigation systems and gravimeters are now routinely used to map the regional gravitational quantities from an aircraft with mGal accuracy and a spatial resolution of a few kilometers. However, airborne gravimeter of this kind is limited by the inaccuracy of the inertial sensor performance, the integrated navigation technique and the kinematic acceleration determination. As the GPS technique developed, the vehicle acceleration determination is no longer the limiting factor in airborne gravity due to the cancellation of the common mode acceleration in differential mode. A new airborne gravimeter taking full advantage of the inertial navigation system is described with improved mechanical design, high precision time synchronization, better thermal control and optimized sensor modeling. Apart from the general usage, the Global Positioning System (GPS) after differentiation is integrated to the inertial navigation system which provides not only more precise altitude information along with the navigation aiding, but also an effective way to calculate the vehicle acceleration. Design description and test results on the performance of the gyroscopes and accelerations will be emphasized. Analysis and discussion of the airborne field test results are also given.

## 1. Introduction

The Earth's gravity is one of the basic forces that affects everything on the earth. It has great importance in geodesy, geodynamics, oceanic science and military usage [1]. The history of the airborne gravimeter could be dated back as early as 1950s [2] and many countries invest lots of money in developing the airborne gravity technique and systems, such as America [3], Russia [4], Canada [5], Germany [6], Denmark [7], *etc.* At present, airborne gravimeters based on StrapDown Inertial Navigation Systems (SDINS) has not been matured enough to be used commercially, e.g., for hydrocarbon exploration. Making use of the SDINS in airborne gravity still has some unsolved problems.

The SDINS is a system of calculating velocity by integration of the total acceleration and computing position by integration of the resultant velocity. The core functional principle of the SDINS is quite simple and concise which comes fundamentally from the Newton's Second Law. Without the knowledge of the gravity anomaly, the navigation algorithm yields an erroneous solution because all the calculation is based on the ellipsoidal vertical [8]. Even with a high-resolution model, position errors will still accumulate to the order of 100 m after one hour of free-inertial navigation [9]. The noise in inertial navigation is the signal in airborne gravimetry, which is the original ideas of Strapdown Airborne Gravimeter (SAG).

The use of a SDINS for airborne gravimetry was pioneered in the early 1990s by researchers at the University of Calgary. The test results showed the feasibility as an accurate and relatively small and low cost alternative to all other available airborne gravity systems [10]. At that time, an accuracy of 2–3 mGal (1 *Gal* = 1 *cm/s*^2^) at a half-wavelength resolution of 5 km was achieved [10]. However, there are no more news of their strapdown gravimeters in recent years. Some other SAGs are under research and getting improved, such as SAGS [11] and Porto system [12]. Improvement of the traditional LaCoste and Romberg gravity meter systems in a strapped-down way showed the feasibility of getting a better accuracy [13]. While improvement takes advantage of the better off-level correction by means of accurate GPS data, the SDINS is a much deeper strapped-down system that the off-level correction depends on the horizontal accelerometers.

Though it has already been proven to be an efficient and accurate way to obtain airborne gravity data, especially over remote regions and those difficult to access, such as the polar and alpine regions of the world [14], the performance of the SDINS is unfortunately affected by long-period errors that severely limit its performance. This implies that an in-depth research on SDINS could fundamentally improve the overall accuracy of such system.

This paper addresses modern gravity survey techniques based on the SDINS with the purpose of yielding a fast and inexpensive airborne gravity system. A SAG system, called SGA-WZ, has already been made by National University of Defense Technology (NUDT). The description of the SGA-WZ is presented with test flight results and comprehensive analysis.

## 2. Principle of Strapdown Airborne Gravimetry

**Figure 1.**
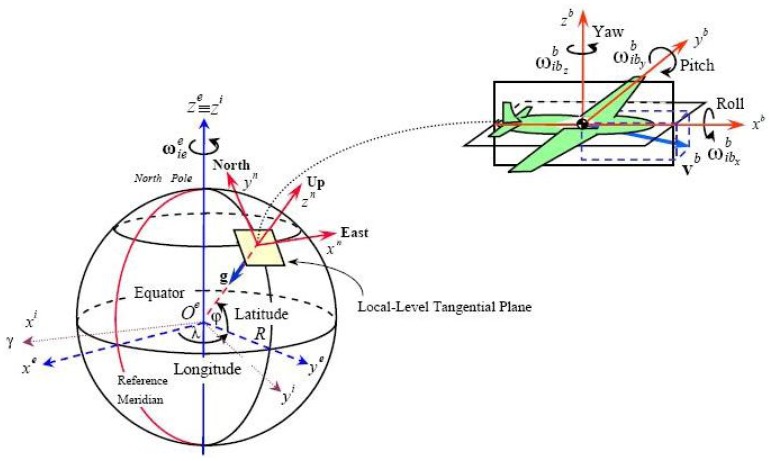
Relationship of different reference frames.

Before the introduction, several reference frames will be presented and their relationship is shown in Figure 1. The inertial frame (i-frame) has its origin at the center of the earth and axes which are non-rotating with respect to the fixed stars, defined by the axes $O_{x}^{i}$, $O_{y}^{i}$ and $O_{z}^{i}$, with $O_{z}^{i}$ coincident with the Earth's polar axis. The Earth frame (e-frame) has its origin at the centre of the Earth and axes which are fixed with respect to the Earth, defined by the axes $O_{x}^{e}$, $O_{y}^{e}$ and $O_{z}^{e}$, with $O_{z}^{e}$ along the Earth's polar axis. The axis $O_{x}^{e}$ lies along the intersection of the plane of the Greenwich meridian with the Earth's equatorial plane, the earth frame rotates with respect to the inertial frame. The navigation frame (n-frame) is a local geographic frame which has its origin at the location of the navigation system point *P* and axes aligned with the directions of north, up and east. The turn rate of the navigation frame with respect to the Earth-fixed frame, $w_{e}^{n}$, is governed by the motion of the point *P* with respect to the Earth. This is often referred to as the transport rate. The body frame (b-frame) is an orthogonal axis set which is aligned with the roll, pitch and yaw axes of the vehicle in which the navigation system is installed.

In airborne gravimetry, the gravity disturbance vector with respect to n-frame *δg^n^* is the goal of the integrated measurement system. It is the difference between the actual gravity vector, *g^n^* and normal gravity *γ^n^* at the same point in space, *δg^n^* = *g^n^* − *γ^n^*. An airborne gravity system measures the deviations of the actual gravity field from the global gravity model which is based on the normal gravity potential. A brief introduction of the principle of the SGA-WZ is given in this section. According to Newton's Second Law, the equation of the vehicle motion with respect to inertial frame (i-frame) could be written as

(1) $$f^{i}={\ddot{r}}^{i}-g^{i}$$

where *r̈^i^* is the acceleration of the vehicle in i-frame, *g^i^* and *f^i^* are the true gravitational acceleration and specific force sensed by the accelerometers respectively. Thus, rearranging the equation yields

(2) $$g^{i}={\ddot{r}}^{i}-f^{i}$$

The specific force *f^i^* is the sensed output of accelerometers which will be transformed into the n-frame. Using the similarity transformation for the angular rates, the equation becomes

(3) $${\dot{\upsilon }}_{e}^{n}=C_{b}^{n}f^{b}-\left(2w_{ie}^{n}+w_{en}^{n}\right)\times \upsilon_{e}^{n}+g^{n}$$

where ${\dot{\upsilon }}_{e}^{n}$ and $\upsilon_{e}^{n}$ is the acceleration and velocity of the vehicle with respect to the Earth, expressed in n-frame, *f^b^* is the specific force measured by the triad of accelerometers in b-frame, $C_{b}^{n}$ is the direct cosine matrix from b-frame to n-frame. *g^n^* is the true gravity vector, $w_{ie}^{n}$ is the rotation rate of the Earth respect to n-frame. $w_{en}^{n}$ is angular rate of the n-frame with respect to the e-frame, expressed in the n-frame. This equation is the basic equation for inertial navigation which provides the basic description of the earth relative velocity evolution in a local level navigation frame. In Equation (3), all the items could be achieved from the GPS except the $C_{b}^{n}f^{b}$. Rearrange the formula yields

(4) $$g^{n}={\dot{\upsilon }}_{e}^{n}+\left(2\Omega_{ie}^{n}+\Omega_{en}^{n}\right)\cdot \upsilon_{e}^{n}-C_{b}^{n}f^{b}$$

where the $\Omega_{ie}^{n}$ and $\Omega_{en}^{n}$ is the skew matrix of $w_{ie}^{n}$ and $w_{en}^{n}$ respectively. Thus, the vertical quantity of the gravity could be written as

(5) $$g_{U}={\dot{\upsilon }}_{U}-(\frac{\upsilon_{E}}{R_{M}+h}+2w_{ie})\text{cos}\phi \cdot \upsilon_{E}-\frac{\upsilon_{N}^{2}}{R_{M}+h}-f_{U}$$

where, *N, U, E* is north, upward and east respect to the n-frame respectively. And the Eötvös correction could be expressed as

(6) $$g_{\text{eot}}=-(\frac{\upsilon_{E}}{R_{M}+h}+2w_{ie})\text{cos}\phi \cdot \upsilon_{E}-\frac{\upsilon_{N}^{2}}{R_{M}+h}$$

where *φ* is the latitude in n-frame, *υ_N_* and * υ_E_* is the north and east velocity of the vehicle with respect to the Earth in n-frame, *R_M_* and *R_N_* are the transverse and meridian radii of curvature respectively.

## 3. Gravimeter Overview

In this section, an overview of the SGA-WZ is given with four subsections including system components, sensor performance, static test and road test. System components subsection gives a description of the system structure and their corresponding function. The ideas of this design interpreted with theoretical explanation is also presented to give a preliminary understanding of the structure. The design structure strongly supports the new idea to fully accomplish the goal of airborne gravimeter. The sensor performance gives the detail introduction of the gravity sensor which would brings us the expected result of SAG and makes a comparison with the other SAGs easier. Static test and road test checks the functional states of the whole system and demonstrates a preliminary feasibility.

### 3.1. System Components

**Figure 2.**
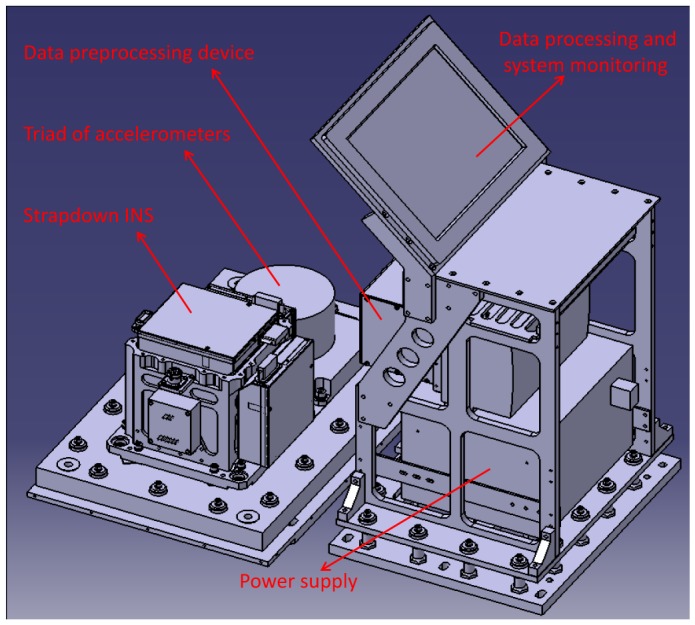
Primary components of the airborne gravimeter SGA-WZ including two cabinets: cabinet of SDINS and cabinet of data recording and system monitoring computer.

The gravimeter is based on a SDINS and Differential GPS (DGPS). The system could be physically divided into two main components: a cabinet containing the SDINS hardware and a triad of accelerometers, and a cabinet containing data recording and system monitoring computer, see Figure 2. The SDINS consists of three navigation-grade Ring Laser Gyroscopes (RLG) and three quartz flexibility accelerometers. These three accelerometers are at a medium accuracy with the bias stability of 1 × 10^−5^ *g*.

Unlike gravimeters used in traditional airborne surveys, the SGA-WZ does not use any spring-type apparatus (like LaCoste & Romberg), and it does not contain any gyro-stabilized inertial platform (like GT-1A, AIRGrav). The whole system is fully strapped to the aircraft and therefore experience the same motion as the aircraft, which requires accelerometers with wide dynamic range and high resolution.

The error of the specific force comes from two aspects, the error of accelerometers and the error of misalignment, as could be described below.

(7) $$\delta f_{sf}=\left[f^{n}\times \right]\cdot \psi +C_{b}^{n}\delta f^{b}$$

where, *δf_sf_* is the error of the specific force, *f^n^* is the specific force with respect to navigation frame (n-frame, north, upward, east), *ψ* is the misalignment angle, $C_{b}^{n}$ is the direct cosine matrix from b-frame to n-frame, *δf^b^* is the bias of the accelerometer. Expanding the first item at the right side of Equation (7) yields:

(8) $$\left[f^{n}\times \right]\cdot \psi =\left[\begin{array}{ccc} 0 & -\psi_{E} & \psi_{U}\\ \psi_{E} & 0 & -\psi_{N}\\ -\psi_{U} & \psi_{N} & 0 \end{array}\right]\left[\begin{array}{c} f_{N}^{n}\\ f_{U}^{n}\\ f_{E}^{n} \end{array}\right]=\left[\begin{array}{c} \psi_{U}f_{E}^{n}-\psi_{E}f_{U}^{n}\\ \psi_{E}f_{N}^{n}-\psi_{N}f_{E}^{n}\\ \psi_{N}f_{U}^{n}-\psi_{U}f_{N}^{n} \end{array}\right]$$

From Equation (8), the misalignment error could be induced into the vertical gravity quantity only on the condition that the horizontal acceleration exists. Given the misalignment at 10 arc seconds, the horizontal acceleration of 0.1g would arose the scale gravity error at 4.8 mGal, which implies that a sufficient attitude could be achieved using medium accelerometers which is mentioned above (1 × 10^−5^*g*). The reason why the SGA-WZ has SDINS and the triad of accelerometers installed side by side is to avoid the high frequent vibration by the dither RLGs, which thus improves the resolution of the accelerometer triad. Therefore, the accelerometers inside the SDINS provide the attitude while the triad of the accelerometers measures the gravity. The advantage of this design is that the accelerometer triad is not affected by the high frequent vibration, which prohibits some inherent error while the SDINS avoids the saturation during strong vertical motion of the aircraft especially the taking off and landing. The data recording is done using PC compatible hardware and software. All inertial data are recorded at a rate of 2 kHz while the GPS data are recorded from a dual frequency receiver at a rate of 2 Hz.

### 3.2. Sensor Performance

Airborne gravity has stringent requirements for the performance of the sensors. These sensors include RLGs and accelerometers which measure the angular motion and translate motion respectively. The performance of the RLGs is shown in the Table 1.

Compared to RLGs, the performance of the accelerometers are more critical to airborne applications. The conflict between wide range and high resolution go through all the design of the SGA-WZ. The performance of the triad of accelerometers without thermal control is given in Table 2.

The statistics is much better when the system is operated in the precise thermal control at a level of 0.02 °*C*. As could be seen in the Table 2, the temperature coefficient is relatively high, which implies that the thermal dependency is one of the key source of systematic errors. As a result, thermal control has been taken into consideration throughout the design. A stability of better than 0.5 mGal/day could be achieved when the system is under the designed thermal control, which is shown in the Section 3.3.

### 3.3. Static Test

**Figure 3.**
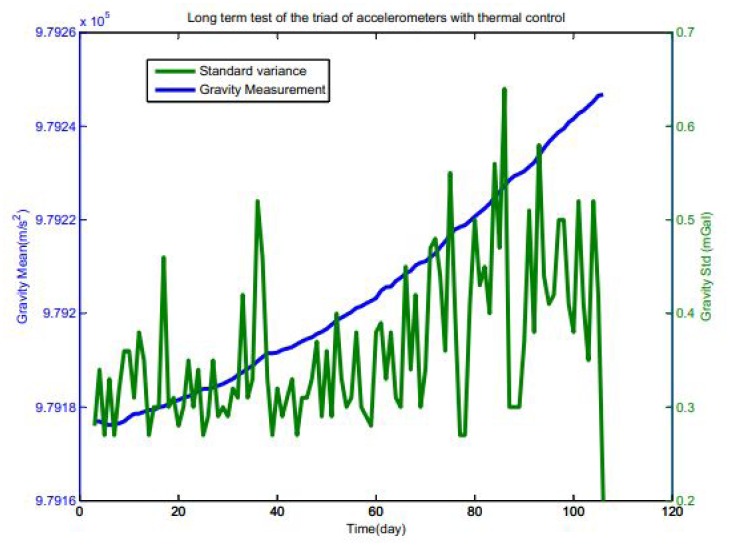
Vertical quantity test results of the triad of accelerometers with thermal control in 104 days.

Test is a necessity to validate the performance after the triad is put together. Systematic test will check out the working state of the whole gravity system. The SGA-WZ, including the sensors, the electronics, the thermal control and other subsystems, was put on a marble platform for 106 days. The results of gravity sensor can be seen in Figure 3. In the figure, the mean and the standard variance of each day is plotted. As indicated, the vertical accelerometer is very stable and varies only 60 mGal in 104 days (In the first two days, the system was disturbed for some personal reason), that is, less than 0.5 mGal/day. The standard variance vary from 0.3 mGal to 0.6 mGal.

### 3.4. Road Test

Airborne flight test is pretty expensive and time consuming whereas road test is an efficient and cheap approach to test the performance of the SGA-WZ in a relative low dynamic cases. This test suggests an average performance of the system drift by comparing two static reference gravity point. During the test, the system was installed in a vehicle and cruised between two gravity reference stations with the distance between the two being more than 30 km. At each reference station the engine was shut down and a duration of 15 minutes was used to collect the data in static. The test was carried out repeatedly three times in one single day with the uninterrupted power supply. Comparison of the seven point measurements at the reference station and the six increments between the references is a validation of the dynamic performance of the SGA-WZ as shown in Table 3. The standard deviation of increments is about 0.45 mGal, which is consistent with the index of static test. Results of the road test suggests the dynamics of the SGA-WZ is good enough for airborne use.

## 4. Primary Problems in System Design

Apart from the sensors used in SGA-WZ, some key problems were taken into serious consideration during the design and construction. There problems mostly have strong relationship either with the theory or with the sensors. Inhibiting systematic errors is the core task of the design.

### 4.1. Precise Thermal Control

The SDINS commonly uses the quartz flexibility accelerometers which should be as accurate as 10^−4^*g* ∼ 10^−5^*g* to meet the requirement of inertial-grade applications. In airborne gravimetry, the long wavelength information is limited by INS accelerometer biases and gyro drifts, while accuracy of the short wavelength information is restricted by the kinematic acceleration errors derived from GPS [15]. For the aim of 1 mGal/1 km airborne gravimeter, the resolution of the accelerometers should be better than 1 mGal at the corresponding bandwidth, which implies that the ordinary quartz accelerometers mentioned above could not be directly made into airborne applications. As stated in [15], for the requirements of geophysical exploration, both the INS and GPS should be improved. The critical factor that affects the accelerometer accuracy is the inherent temperature sensitivity, which highlights the importance of the thermal control systems. The temperature is set at a constant independent with the outside environment. Regarding different climates the system will undergo, the temperature should be kept at a stability of better than ±0.02 °*C* according to the accelerometers we used. The main reason for ±0.02 °*C* is to make sure the temperature error would then be well below 1 mGal. Redundancy is also considered to prohibit other temperature related errors.

### 4.2. System Damping

In strapdown cases, all kinds of dynamic motions as well as the aircraft engines cause high frequency noise, which consequently affects the accuracy of the gravity sensors severely. Without any mechanical platforms like gyro-stabilized or inertial platforms, the SAGs will go through much more severe influence in the dynamic flights. Therefore, well-designed damping system with sufficient performance becomes more critical than the traditional gravity meters. The damping system used in SGA-WZ is the shock absorber installed between the system and the aircraft. Two aspects of the damping system were taken into account: the cut frequency and the stiffness. Simulation on different damping design of the SGA-WZ has been carried out and verification was taken on the field test flights. Although the preliminary results showed the feasibility of current design, the damping system is more than urgent to be updated. A thorough analysis and experiment on the damping system will be greatly beneficial to the SAGs, which is already in the design of the next generation.

### 4.3. Integrated Navigation Technology

The error of the measurement of the specific force comes mainly from the accelerometers and the attitude misalignment. Making use of the complementary characteristics of SDINS and DGPS can offer a significantly increased accuracy on the attitude estimation, which therefore improves the accuracy of the specific force measurement. The integration, generally, exploits the Kalman filtering technology which optimally estimate the navigation parameters and the sensor error models. The problem is in what condition could the integration figure out the optimal estimation. The straight heading and the constant speed, however, are not a favorable condition for the Kalman filtering and the tuning is time-consuming. The estimation has a direction impact on the alignment. Integration navigation could be regarded as somehow an alignment with the purpose of ensuring that the gravity could be projected correctly and precisely. Furthermore, the bias of the accelerometers is strongly coupled with the attitude, which implies that a good estimation makes a good alignment. Others problems such as time synchronization, low passing filtering, lever arm effect compensation and vehicle acceleration determination are of great importance, besides of those discussed above.

## 5. Flight Results and Analysis

**Figure 4.**
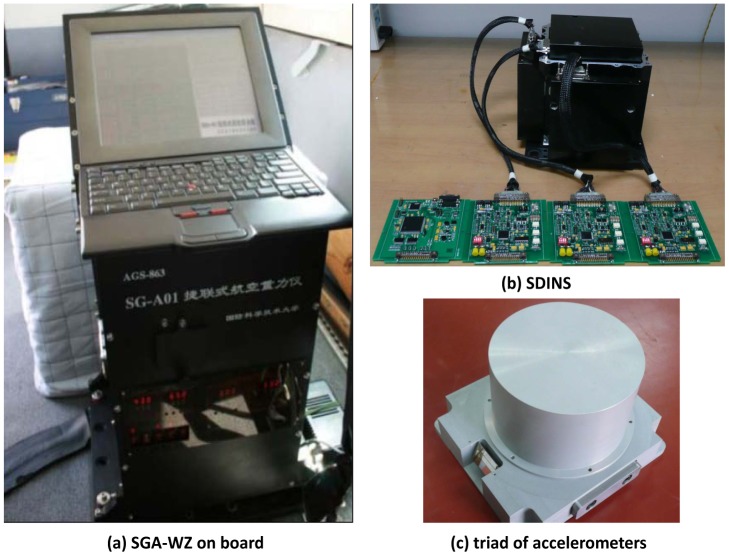
The SGA-WZ mounted on the aircraft and its primary components.

**Figure 5.**
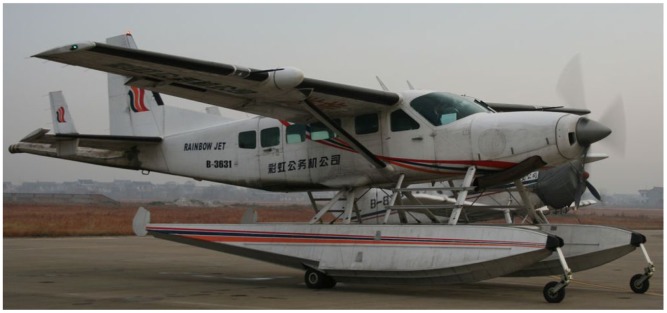
The single engine small aircraft Cessna 208B Grand Caravan with the engine running (Taken in Shandong in April 2010).

Flight tests of SGA-WZ prototype system have already been carried out three times, *i.e.*, in Jiangsu province in December 2007, Shandong province in April 2009 and Shandong province in April 2010, respectively. During all the flight tests, SGA-WZ was installed in a Cessna 208B Grand Caravan, a single engine aircraft, as Figure 4 shows. This aircraft has the advantage to be able to fly safely at a low speed and low dynamics. A picture of the Cessna 208B Grand Caravan with the engine on is provided in Figure 5. No additional data acquisition and logging device is required because this is accomplished by the SGA-WZ itself.

Among the three flight tests, the first two tests were not a success because of several technical problems. Each time, hardware upgrade and software improvements were made to gradually adapt to the airborne gravity requirements. The third flight test showed relatively satisfying results which are described here. The flight test began on 2 April 2010 and lasted until 15 May 2010. During this period, SGA-WZ was on power without interruption. Flights were made at a nominal altitude of 400 m and at a speed of about 60 m/s. For most of the time, the aircraft were controlled by the autopilot.

In order to calculate the vehicle acceleration by double difference of the GPS position, a GPS antenna was mounted on the top of the aircraft while two GPS reference stations were established with the coordinates precisely calibrated. One of reference station was on the roof of one building within the airport while the other was located in the airport field. Both reference stations were equipped with NovAtel Millennium receivers.

**Figure 6.**
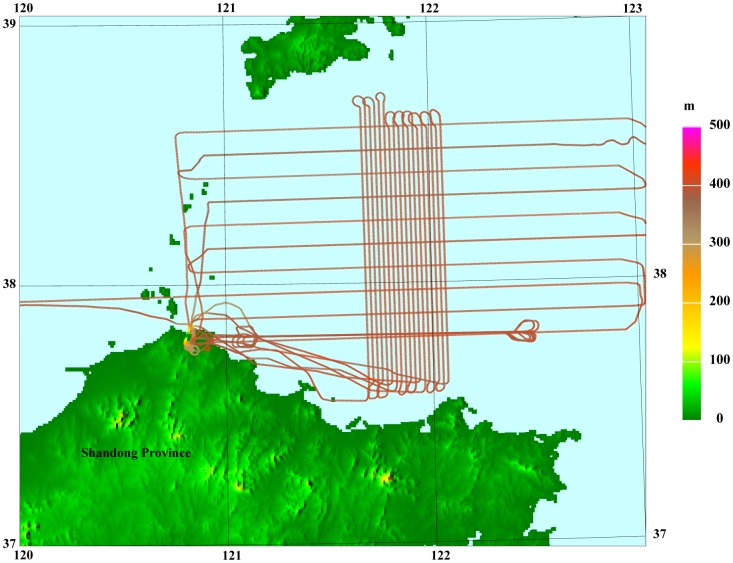
The map of the flight test region carried out in April 2010.

The map of the flight test were shown in Figure 6 as well as the flight lines. It has one repeated line, ten control lines and eighteen survey lines. The control lines is along the east-west while the survey lines along the north-south.

Several programs were made to process the data. These programs covered several steps. First, it processes the carrier phase information from the dual frequency GPS receivers and figures out the accurate position. Double difference of the position yields the velocity and acceleration of the vehicle. After the lever arm compensation, these data are put into the Kalman filter to carry out the integrated navigation as the observation. The resultant output of the Kalman filter is the navigation parameters and the specific force measured by the triad of the accelerometers which was under special designed thermal control. The discrepancy of the specific force and vehicle acceleration results in the gravity disturbance, which was further put into the low-pass filter. Most of the high frequency noise such as the engine noise, the turbulence and the dithering noise of the RLG will be filtered by the low-pass FIR filter.

**Figure 7.**
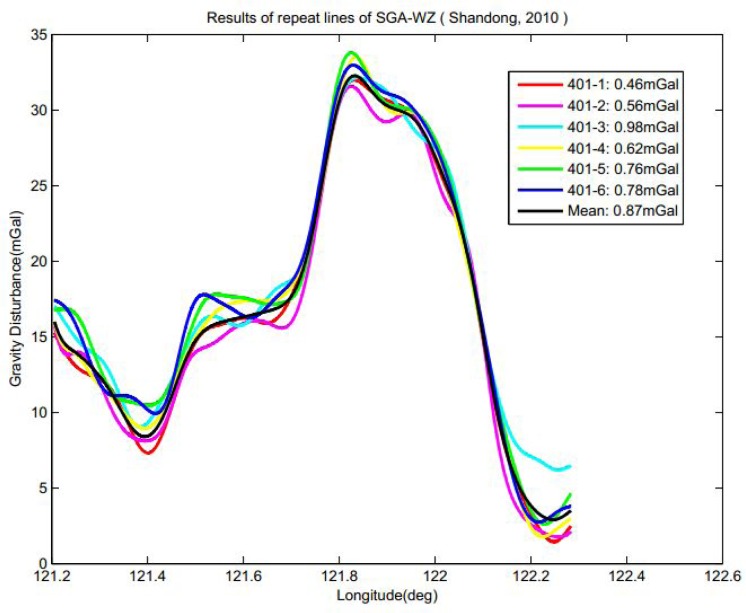
Result of the repeat lines of SGA-WZ in the flight test in Shandong in April 2010.

Due to lack of ground data, the evaluation of the flight test is carried out by checking the performance of the repeated lines and the statistics of the crossover points in the grid flights. Preliminary results could be seen in Figure 7. As can be seen in the figure, for the low-pass filter period 200 s, the internal consistent accuracy of repeated lines can be better than 0.87 mGal for a spatial resolution of 6.0 km in the means of standard variance. However, systematic error severely affects the result with the level of 1.1 mGal, which makes the RMS accuracy of nearly 2 mGal. In particular, the difference of the mean value between every single line has not been identified yet. Further, some unknown random noise is markedly clear in Figure 7, which makes the figure somehow out of accordance. These errors would be reduced by further filtering at the expense of losing resolution. More investigation is ongoing in order to identify the sources of these systematic errors and feasible methods for compensation.

## 6. Conclusions

In this paper, the basic principle, key technology and test results of the SGA-WZ is presented. A gravity anomaly estimation better than 2 mGal for a half wavelength at 6.0 km was delivered by the SGA-WZ. Results show that it is a very appropriate device for airborne gravimetric surveys in a standard geophysical survey aircraft. More flight tests should be carried out to test the tolerance of the aircraft dynamics to prove its ability and accuracy for drape flights. Preliminary analysis of the systematic error demonstrates that the gravity sensor drift in dynamic flights is the dominant error source. Other errors are the effect of the earth magnetic field, the misalignment estimation and the error model of the accelerometers.

## Figures and Tables

**Table 1.** The performance of the RLGs.

**Bias** (°***/h***)			**Scale factor**		
	
	**Stability**	**Repeatability**	**Repeatability**(**1 *σ***)	**asymmetry** (3***σ***)	**nonlinearity** (**1*σ***)
X	0.0033	0.00044	8.7E-07	6.4E-06	1.08E-06
Y	0.0032	0.00038	8.5E-07	1.7E-06	1.70E-06
Z	0.0029	0.00157	1.1E-06	4.6E-06	1.18E-05

**Table 2.** The performance of the accelerometers.

**item**	**X**	**Y**	**Z**
range (g)	±10	±10	±10
Bias (*μg*)	8.1	9.7	2.8
Scale Factor (mA/g)	1.344	1.267	1.345
2nd Non-linear coefficient (*μg/g*^2^)	−7.9	7.4	−12.9
Scale Factor Stability(ppm)	23.3	19.9	5.5
Temperature coefficient(*μg/*°*C*)	8.4	−3.6	11.5
Stability in 4 hours (*μg*)	1.9	1.4	2.5

**Table 3.** Result of road test of SGA-WZ.

**Reference Station**	**Gravity(mGal)**	**Increment(mGal)**		
	
Point A	979161.78		mean	std
Point B	979143.28	−18.55		
Point A	979160.98	17.33		
Point B	979143.65	−17.74	−17.845	0.450
Point A	979161.84	17.75		
Point B	979144.10	−18.19		
Point A	979161.61	17.51		
